# A five-year retrospective study focused on urinary tract infections in kidney transplant recipients in the current era of immunosuppression

**DOI:** 10.3389/fmed.2025.1606224

**Published:** 2025-07-22

**Authors:** Jorge Andrade-Sierra, Jorge Carlos Andrade-Martínez, Elsa Angélica Fuentes-López, Enrique Rojas-Campos, Víctor Martínez-Mejía, Eduardo González-Espinoza, Ernesto German Cardona-Muñoz, José Ignacio Cerrillos-Gutiérrez, Luis Alberto Evangelista-Carrillo, Miguel Medina-Pérez, Moisés Cruz-Landino, Adriana Banda-López, Alejandra Guillermina Miranda-Díaz, J. Ahuixotl Gutiérrez Aceves, Jorge Andrade-Ortega, Kevin Javier Arellano-Arteaga, Antonio de Jesús Andrade-Ortega, Laura Elizabeth Aguilar Fletes, Gerardo González-Correa, Priscila Preciado, Joel E. Verdugo-Correa, Héctor Leonardo Pazarín-Villaseñor, Ana Luisa Corona-Nakamura, Mauricio Carvallo-Venegas

**Affiliations:** ^1^Department of Nephrology and Organ Transplant Unit, Specialties Hospital, National Western Medical Centre, Mexican Institute of Social Security, Guadalajara, Mexico; ^2^Department of Physiology, University Health Sciences Center, University of Guadalajara, Guadalajara, Mexico; ^3^Medical Research Unit in Renal Diseases, Specialties Hospital, National Western Medical Centre, Mexican Institute of Social Security, Guadalajara, Mexico; ^4^Department of Internal Medicine, Hospital Civil de Guadalajara “Dr. Juan I. Menchaca”, Guadalajara, Mexico; ^5^Department of Infectology, Specialties Hospital, National Western Medical Centre, Mexican Institute of Social Security, Guadalajara, Mexico

**Keywords:** urinary tract infection (UTI), kidney function, acute kidney injury (AKI), immunosupresion, kidney tranplantation

## Abstract

After kidney transplantation, UTI are the most common infection concern and can cause acute renal injury (AKI) in allografts. However, long-term allograft function, loss, and mortality risk are inconsistent. A retrospective cohort research of 1,341 kidney transplant recipients (KTR) from January 2014 to March 2019 assessed UTI incidence, risk factors, and consequences on AKI and allograft function in the first year. All first-year post-transplant UTI patients were recorded. Third-generation cephalosporin (1 gr, two doses) and 500 mg intravesical amikacin were given to all patients 1 day before surgery. After that, patients had TMP-SMX (160/800 mg qd) for 3–4 months to prevent Pneumocystis jirovecii pneumonia, and the main immunosuppressive regimen was mycophenolate mofetil, prednisone and a Calcineurin inhibitors. The UTI incidence was 42.5%. *Escherichia coli* was the most common causal bacteria, accounting for a significant amount of strains of Extended-spectrum beta-lactamase (ESBL) and AKI occurred more in the first and second UTI. Our analysis showed risk factors of anti-thymocyte globulin (ATG) use (RR 1.52; *p =* 0.032), double J catheter (RR 1.9; *p =* 0.004), and urinary tract abnormalities (RR 1.92; *p =* 0.007). Although UTI was common and associated with AKI, it did not affect allograft function at 12 months post-transplantation.

## Introduction

Urinary tract infection (UTI) is the most common infectious complication in kidney transplant recipients (KTR) (1–4), with a cumulative incidence of up to 53.7% during the first year (4), and prevalence rates ranging from 7.3 to 75% (5). Lack of uniformity in the diagnostic criteria, immunosuppression variability, and differences in the use of antimicrobial prophylaxis can explain the variation in prevalence (5–7). Known risk factors include: older age, female sex, obesity, diabetes, urinary tract abnormalities, systemic diseases, deceased kidney donor, time on dialysis, use of a urethral stent, allograft dysfunction, hepatitis C infection, cytomegalovirus (CMV) infection, over-immunosuppression, and chronic kidney disease (CKD) itself, which leads to functional alterations in the urinary tract (such as reduced antibacterial properties of urine, loss of the urothelial protective mucosa, and immunological changes associated with uremia) (5, 8–14). Gram-negative microorganisms account for more than 70% of UTIs, with *Escherichia coli* (*E-coli*) being the most common pathogen (30–80%) (5, 10, 15, 16). A challenging, unfortunate problem with UTIs is the presence of multi-drug resistant (MDR) infections, which arise due to the inadequate use of prophylaxis or treatment of asymptomatic bacteriuria (AB), allograft dysfunction, and the presentation of recurrent and nosocomial UTI (17, 18). It is currently estimated that one in every ten KTR develops a UTI caused by an Extended-spectrum beta-lactamase producing *Enterobacteriaceae (ESBL-PE),* carrying a risk of recurrence three times greater with a clinical impact of causing acute kidney injury (AKI) that can lead to long-term changes in allograft function, allograft loss, and high mortality; although underlying causes remain unclear (19–29).

## Patients and methods

A retrospective cohort study included 1,341 kidney transplant recipients from January 2014 to March 2019, conducted in the Transplant Division of the Specialties Hospital at the National Western Medical Center of the Mexican Institute of Social Security in Guadalajara, Jalisco, Mexico. All patients receiving kidney transplantation from a living (related or known) or deceased donor, with any immunosuppressive regimen, were included and followed up for a minimum of 1 year (monitoring every week for 1 month, every 15 days for the subsequent 3 months, and then monthly for 8 months). Multiorgan recipients, any patients who lost their graft immediately due to surgical complications or hyperacute rejection within the first week post-transplantation, those with primary non-function of the allograft, patients who died within the first month after transplant, and those with incomplete information in the electronic records during the follow-up year were not included in this cohort. Conventional prophylaxis, according to our hospital attention protocol, was 500 mg intravesical amikacin prior to surgery, which was administered to all patients, plus a third generation cephalosporin (cefotaxime 1 g IV or ceftriaxone 1 gr IV, two doses each) prior to surgery. All patients received prophylactic dose of 160 mg trimethoprim and 800 mg sulfamethoxazole qd for 3 to 4 months post-transplantation to prevent *Pneumocystis jirovecii* pneumonia.

The following data were collected: recipient’s age, sex, history of diabetes mellitus or hypertension, anthropometric characteristics, anatomical alterations in the pre-transplant urinary tract, non-functioning native kidneys, residual uresis, etiology of chronic kidney disease, type and duration of kidney replacement therapy, duration of bladder catheterization, or indwelling urethral catheter, immunosuppression (induction, maintenance and desensitization therapies) and number of bacterial UTI.

The presence of post-transplantation UTIs was documented by clinical evaluation (dysuria, urinary urgency, pain over allograft, chills or fever), pathological urinalysis and systemic inflammatory response with confirmation by urine culture, were registered. In relation to taking urine cultures, our protocol is as follows: after antisepsis of the perineum and/or glands with sterile materials, a mid-stream sample in a sterile receptacle is obtained for the analysis, and the culture medium used was Blood Agar and Mac-Conkey Agar. As per our hospital protocol, in the case of clinical suspicion of complications, all patients undergo ultrasound or kidney tomography to rule out complications (obstruction or collections), all of which were recorded. Urodynamic studies were not registered in any patient. Antibiotic treatment, as well as modifications and duration of treatment, were recorded. Lastly, the serum creatinine (SCr) levels that were determined during the patient’s routine follow-up and during each episode of UTI were recorded.

### Definitions

We aimed to adhere to current UTI guidelines and considered the following definitions, although these definitions were adjusted depending on the information obtained from patient records or in the electronic files. Additionally, we excluded AB from this analysis.

#### Urinary tract infections (UTI)

Urinary tract infections defined by clinical evaluation (dysuria, urinary urgency, pain over allograft, chills or fever) and pathological urinalysis; or, as the presence of bacteria in the urine with ≥10^5^ CFU/mL in the presence of local and/or systemic signs or symptoms of infection. All UTIs that were uncomplicated (dysuria, frequency, or urinary urgency and the absence of fever or pain over allograft) and complicated (fever or bacteremia with one or more of the following symptoms: pain over allograft, lumbar pain, chills) were registered and analyzed concurrently, and we did not differentiate between lower and upper UTI.

#### Recurrent urinary tract infections (RUTI)

Recurrent urinary tract infections defined as (≥2 UTI episodes per year) new episodes of infection in the same patient, with or without bacterial isolation in a culture, obtained two to 4 weeks after the completion of the previous treatment.

#### Acute kidney injury (AKI)

Acute kidney injury defined solely as an increase of more than 30% in SCr compared to baseline values during the UTIs episode infections.

### Immunosuppression characteristics

Immunosuppression management was carried out according to the protocols at our center: immunosuppression induction was based on basiliximab (BSL) 20 mg at 0- and 4-days post-transplant, or anti-thymocyte globulin (ATG) at a dose of 1 mg/kg/day (accumulated dosage 4 mg/kg).

Maintenance immunosuppression was based on mycophenolate mofetil (MMF) 1.5 to 2 g/day, tacrolimus (TAC) 0.12 mg/Kg/day or cyclosporine A (CsA) 4 mg/Kg/day (with dose adjustment according to serum levels), and prednisone (PDN) of 1 mg/kg/day starting from transplantation, with a reduction in dose to achieve 0.1 mg/kg/day by the third post-transplant month. Desensitization therapies before transplantation were conducted according to protocol, with three plasma exchanges (plasmapheresis) and/or immunoglobulin at 200 mg/kg/day administered on alternating days, along with rituximab at 375 mg/m^2^ (one or two doses with a 15-day interval), or immunoglobulin at 200 mg/kg/day for 3 days and rituximab at 375 mg/m^2^, based on medical discretion.

### Statistical analysis

Data are presented as mean ± standard deviation or median (percentiles 25–75%), and numbers and percentages where appropriate. Chi-squared test or Fisher’s exact test were used to compare proportions. The unpaired Student *t*-test was used to compare continuous variables between groups, and the Mann–Whitney U-test was used to compare continuous variables with non-normal distribution. Logistic regression was applied individually to each variable. All episodes of UTIs were modeled in the logistic regression. Statistically significant variables in the univariate analysis were introduced in a multivariate model based on forward stepwise logistic regression to identify independent risk factors for UTI. Associations are represented using relative risk (RR) with a 95% confidence interval (95% CI). All results with a value of *p <* 0.05 were considered statistically significant. Statistical analysis was performed with SPSS™ software, version 22 (SPSS, Inc., Chicago, IL).

### Ethical considerations

The present research complies with the Ethical Principles for Medical Research in Human Beings as stipulated in the Declaration of Helsinki 64th General Assembly, Fortaleza, Brazil, October 2013; in addition to adhering to the standards of good clinical practices. All operations followed the General Health Legal Guidelines for Health Care Research in Mexico, 2nd Title, in Ethical Aspects for Human Research in Human Beings, Chapter 1, Article 17. All patients signed the informed consent forms in the presence of witnesses. The local Ethics and Research Committee accepted the study (R-2017-1301-103).

## Results

A total of 1,341 recipients were included in this cohort with a minimum follow-up of 1 year. During the study period, 556 patients (42.5%) developed at least one episode of bacterial UTI: a first episode occurred in 523 patients (39%), a second episode in 241 patients (18%), and a third episode in 121 patients (9%). Based on whether or not they developed a UTI, patients were divided into two groups, the characteristics of which are presented in Table 1. Most patients were young, male, and received their first transplant from a living donor. The incidence of UTI was higher in recipients with a history of diabetes mellitus and pre-transplant anatomical abnormalities of the urinary tract (Table 1). Occurrence of UTI was more prevalent in patients using a double-J catheter and in those who had a Foley catheter for five or more days. A total of 88.4% received maintenance immunosuppression with TAC/MMF/PDN, and 62% underwent induction therapy with ATG (see Table 2).

**Table 1:** Demographic characteristics.

Characteristics	Total *n* = 1,341	UTI *n* = 556 (41.5)	No UTI *n* = 785 (58.5)	*p*
Age-recipients (years)	31 ± 11	32 ± 12*	30 ± 10	0.005*
Sex-recipients- Male, *n* (%)	926 (69)	352 (63.3)*	574 (73.1)	0.001*
Etiology CKD (%)				
Unknown	1,137 (84.8)	446 (80.2)	691 (88)	0.005*
Diabetes	92 (6.9)	51 (9.9)*	41 (5.2)	
Glomerulopathies	54 (4.0)	30 (5.4)	24 (3)	
Polycystic Kidney disease	23 (1.7)	10 (1.8)	13 (1.7)	
Others	35 (2.6)	19 (3.4)	16 (2)	
Comorbidity (%)				
None	138 (10.3)	55 (9.9)	83 (10.6)	NS
Diabetes	24 (1.8)	12 (2.1)	12 (1.5)	
Diabetes/Hypertension	68 (5.1)	39 (7)	29 (3.7)	
Hypertension	1,111 (82.5)	450 (81)	661 (84.2)	
Pre-transplant diuresis (%)				
Anuria	99 (7)	37 (6.7)	62 (7.9)	NS
>100–1,000 mL	963(72)	407 (73.2)	556 (70.8)	
>1,000 ml	279 (21)	112 (20.1)	167 (21.3)	
Pre-transplant anatomical alterations *n* (%)				
No	1,234 (92)	468 (84.2)	766 (97.6)	0.001*
Yes	107 (8)	88 (15.8)*	19 (2.4)	
Replacement therapy *n* (%)				
PD	433 (32.3)	159 (28.6)	274 (34.9)	0.05*
HD	863 (64.3)	378 (68)	485 (61.8)	
Anticipated	45 (3.4)	19 (3.4)	26 (3.3)	
Time on dialysis *n* (%)	46 ± 40	47 ± 40	45 ± 40	NS

*p < 0.05 UTI vs. No UTI.

**Table 2:** Transplant characteristics.

Characteristics	Total *n* = 1,341	UTI *n* = 556 (41.5)	No UTI *n* = 785 (58.5)	*p*
Donor type (%)				
Living donor	1,152 (85.9)	469 (84.4)	683 (87)	NS
Deceased donor	189 (14.1)	87 (15.6)	102 (13)	
Transplant number, *n* (%)				
First	1,267(94.5)	511 (92)	756 (96)	0.001*
Second	74 (5.5)	45 (8)	29 (4)	
Desensitization *n* (%)				
Yes	49 (3.7)	35 (6.3)	14 (1.8)	0.001*
No	1,292 (96.3)	521 (93.7)	771 (98.2)	
Induction, *n* (%)				
Thymoglobulin	838 (62.4)	335 (60)	503 (64)	NS
Basiliximab	503 (37.5)	221 (40)	282 (36)	
Time with Foley catheter, *n* (%)				
=5 days	992 (74)	372 (67)	620 (79)	0.001*
>5 days	349 (26)	184 (33)*	165 (21)	
Double J catheter, *n* (%)	249 (18)	129 (23)*	120 (15)	0.002*

*p < 0.05 IVU vs. No IVU.

We found that AKI occurred in 54.6% of patients who experienced at least one episode of UTI, with the majority of cases occurring in the first month post transplantation. Both the first and second UTI episode, 52 and 3%, occurred at 30 days and were related with AKI (Table 3) The most frequently isolated infectious pathogen in the first two UTI episodes was *E. coli*, with a significant percentage of Extended-spectrum beta-lactamase (ESBL) strains (Table 4). During the first UTI episode, *E. coli* was isolated in 51% of cases (22.5% Spp. and 29% *ESBL*), while *E. faecalis* was identified in 21.6% of cases (Table 4). According to our center’s antimicrobial susceptibility patterns, carbapenems were used to treat the first and second episodes in 75.4 and 67.8% of cases, respectively and due to the detection of multi-resistant bacteria (*Pseudomonas* spp., *E. faecalis*, *A. baumanni*, etc.) and the severity of the cases, piperacillin/tazobactam was used in the third episode at 54.7% compared to 26.5% for carbapenems. Most recorded bacteria were resistant to quinolones and trimethoprim/sulfamethoxazole. Anatomical abnormalities [RR 1.92, 95% confidence interval (CI) 1.30–3.0], double J catheter (RR 1.9, 95% CI 1.93–2.91), and the use of ATG, were independent variables associated with at least one episode of bacterial UTI (Table 5). At the end of follow up, kidney allograft function was evaluated solely based on SCr; according to the presence, or not, of UTI, and no significant differences were noted. In individuals with a single UTI, SCr levels were higher at 12 months (Tables 6, 7). Urological complications (urethral stenosis, urethral obstruction, and urinary leakage) were recorded with low frequency in the present cohort, which made their analysis unfeasible.

**Table 3:** Urinary tract infection episodes and their association with acute kidney injury (AKI).

Infectious episodes		AKI	No AKI	*p*
Total *n* (%)	556	304 (54.6)	252 (45.3)	0.001*
1° Episode, *n* (%)	523	290 (55.4)	233 (44.6)	0.001*
2° Episode, *n* (%)	241	133 (55.2)	108 (44.8)	0.001*
3° Episode, *n* (%)	121	69 (57)	52 (43)	NS

* Means that it is a significant *p*-value.

**Table 4:** Frequency of microorganisms isolated in urine cultures.

Urine culture, *n* (%)	1°	2°	3°
	*n* = 523 (39)	*n* = 241 (18)	*n* = 121 (9)
Negative	52 (10)	34 (2.5)	19 (1.4)
*E. coli* spp.	118 (22.5)	76 (5.7)	10 (0.7)
*E. coli* ESBL	151 (29)	108 (8.1)	43 (3.2)
*A. baumanii*	10 (2)	–	15 (1.1)
*K. pneumoniae*	53 (10)	20 (1.5)	12 (0.9)
*P. aeruginosa*	13 (2.5)	–	18 (1.3)
*E. faecalis*	113 (21.6)	1 (0.1)	–
*Candida albicans*	9 (1.7)	–	–
*K. oxitoca* ESBL	5 (1.0)	–	–

**Table 5:** Logistics regression analysis showing variables associated with at least one episode of bacterial UTI.

Variable	Multivarate analysis		
	RR	CI 95%	*p*
Induction-ATG	1.52	1.04–2.2	0.032
Double J catheter	1.90	1.23–2.91	0.004
Urinary tract abnormalities	1.92	1.30–3.0	0.007

**Table 6:** Kidney function according to the presence of UTI.

Characteristics	Total *n* = 1,341	UTI *n* = 556 (41.5)	No UTI *n* = 785 (58.5)	*p*
Creatinine baseline, (mg/dL)	1.07 ± 0.64	0.99 ± 0.4	1.1 ± 0.8	0.0001*
Creatinine, at 1 year (mg/dL)	1.20 ± 1.0	1.16 ± 0.8	1.21 ± 1.17	NS

* Means that it is a significant *p*-value.

**Table 7:** Renal function according to the number of UTI episodes.

Characteristics	Total *n* = 556	UTI = 1° *n* = 308 (55.4)	UTI >2 *n* = 248 (44.6)	*p*
Baseline creatinine, (mg/dL)	1.07 ± 0.64	0.97 ± 0.36	0.99 ± 0.38	NS
Creatinine at 1 year (mg/dL)	1.19 ± 1.02	1.20 ± 1.02*	1.11 ± 0.34*	0.033*

* Means that it is a significant *p*-value.

## Discussion

UTI is one of the most prevalent infections during the post-transplant period, leading to undesirable outcomes, especially due to its association with AKI (27, 30), which contributes to reduced kidney function over time, lower allograft survival rates, and increased mortality (20–29).

In our study, the incidence of UTIs among patients with at least one episode was 41.5%, whereas it was 39% for those with only a single first episode, consistent with prior reports (4, 5, 12, 16, 23, 25, 31). Similarly, Arabi et al. (25) reported 35%, in patients followed for just 6 months. The timing of the initial UTI episode in our cohort (within the first 30 days post-transplant) aligns with previous research (12, 24, 25, 32).

Although some studies report similar or higher UTI rates, these often involve older patients, deceased donors, or cases with delayed graft function —factors known to increase incidence (29, 31, 33). In contrast, our cohort included only 15% deceased donors and did not report delayed graft function, making direct risk assessment challenging.

Notably, compared to other young populations receiving living donor transplants, such as those described by Khedr et al. (34), who reported a 27.3% incidence, our study found a higher rate of UTIs; however, their analysis was limited by a small sample size and inclusion of other infection types.

It is noteworthy that, 63.3% of UTI cases in our cohort occurred in male patients, which contrasts with previous studies that have identified female sex as a significant risk factor for UTI (5, 12, 16, 24, 25, 31). Beyond sex-related differences, several other risk factors for UTIs in KRT have been identified. For example the diabetic nephropathy exhibited a higher incidence of UTI in our cohort, consistent with previous studies identifying diabetes and diabetic nephropathy as risk factors (5, 14, 35).

However, diabetic nephropathy was not found to be an independent predictor in our analysis.

*Urinary tract abnormalities* are a well-established risk factor for UTI (5, 25), as evidenced by their association with at least one UTI episode when considered collectively in our cohort, although individual pathologies could not be analyzed due to the small sample size.

In our center, the use of a double J catheter—another documented risk factor for UTI. — (5, 25, 36, 37) is reserved for cases where urinary tract abnormalities or perioperative anatomical complexity, to prevent major urological complications such as urinary leakage or obstruction. Although less than a quarter of patients received a double J catheter, this was a significant risk predictor (RR 1.90; CI: 1.23–2.91, *p* < 0.004).

The literature indicates that early (14–21 days) (25, 36, 38–40), or even very early (<7 days) (41), removal of the double J catheter reduces infection risk without significantly increasing urological complications, in our center we opted for delayed removal (6–8 weeks) due to the specific clinical indications that initially warranted catheter placement, prioritizing the prevention of major urological complications in these complex cases.

Regarding Foley catheters, although previous studies have not found significant differences in UTI rates based on early or prolonged removal (42, 43). In our cohort we observed a higher incidence of UTIs with long-term use (>5 days). However, our analysis did not identify this as a significant risk factor, in contrast to other reports (5).

On the other hand, it is recognized that intensified immunosuppressive therapy increases UTIs. Although ATG is increasingly used in higher-risk or deceased donor transplants due to its efficacy in reducing AR (44), it does not improve outcomes in living donor transplants and increases viral and bacterial infections (13, 45, 46).

Although some studies do not consistently identify BSL or ATG as risk factors for UTIs (4, 16, 22, 25, 34), other research reports a higher risk associated with ATG (13, 45, 47). In a cohort study conducted at our institution, we previously established an association between the development of CMV and low-dose ATG (48).

Now, compared to BSL, we still observe a significant rate of UTI, supporting research showing that higher ATG accumulation increases the incidence of UTIs.

Li, S. et al., found that UTI incidence dropped to 5% from 19.8% when ATG accumulation was less than 6.34 mg/kg (13). At our center found that even with, low doses (4 mg) had more UTIs than BSL.

A significant aspect of UTI is its association with AKI, which can lead to allograft loss and increased mortality in KTR (27, 49, 50). In our cohort, over 50% of UTIs were linked to AKI, consistent with previous reports (27, 30, 51).

Notably, we did not differentiate between urosepsis and other complicated UTIs, which may explain the high incidence observed.

While the long-term impact of UTI-related allograft dysfunction remains debated—some studies report no significant deterioration, while others observe worse outcomes (19–26, 28)—we did not find evidence of allograft function decline at follow-up, even among patients with recurrent UTIs similar to other reports (20, 22, 23, 52–54), likely due to prompt antibiotic treatment.

While Sánchez et al. (53), observed a negative effect—particularly when analyzing complicated and recurrent UTIs—other studies, such as Ariza-Heredia et al. (20), found no significant difference in kidney function between recipients with or without UTI, although a tendency toward graft deterioration was noted when radioisotope were used.

This highlights the ongoing controversy regarding the long-term consequences of UTIs on graft outcomes.

Lastly, although rising antibiotic resistance is a concern, our center’s use of carbapenems is justified by local bacterial sensitivity and rapid patient recovery, including from AKI.

Limited pathogen data prevented recurrence analysis, but the frequent use of carbapenems underscores the need for ongoing monitoring and prevention strategies against multidrug-resistant bacteria.

Our research has some limitations: this retrospective study was single-center. Both upper and lower urinary tract infections and complicated (urosepsis) and simple UTI episodes were not distinguished, which could have affected AKI incidence and kidney function results. A lack of long-term follow-up and histopathology data (protocol biopsies) limited the study’s capacity to objectively assess the influence of UTIs on AR and chronic allograft damage.

Despite these limitations, the huge sample size and important UTI epidemiology data from Mexico’s (55) most active kidney transplant referral facility make our study strong.

In conclusion, this study demonstrates a high incidence of UTIs among KTR, significantly associated with the development of AKI. However, no deterioration in allograft function was observed at the end of follow-up. Over-immunosuppression with ATG, the presence of a double-J catheter, and pre-existing urinary tract abnormalities were confirmed as key risk factors for UTI.

These findings highlight the need for long-term monitoring to better understand the true impact of UTIs on graft function and patient outcomes.

## Data Availability

The raw data supporting the conclusions of this article will be made available by the authors, without undue reservation.

## Glossary

UTI: Urinary tract infections
KTR: Kidney transplant recipients
CMV: Cytomegalovirus
CKD: Chronic kidney disease
E-coli: *Escherichia coli*
MDR: Multi-drug resistance
AB: Asymptomatic bacteriuria
CFU: Colony-forming units
ESBL-PE: Extended-spectrum beta-lactamase producing Enterobacteriaceae
AKI: Acute kidney injury
SCr: Serum creatinine
RUTI: Recurrent urinary tract infections
BSL: Basiliximab
ATG: Thymoglobulin
MMF: Mycophenolate mofetil
TAC: Tacrolimus
CsA: Cyclosporine A
PDN: Prednisone
ESBL: Extended-spectrum beta-lactamase
AR: Acute rejection
