# Supplementary material for: A five-year retrospective study focused on urinary tract infections in kidney transplant recipients in the current era of immunosuppression
Source: Front Med (Lausanne). 2025 Jul 22;12:1606224. doi: 10.3389/fmed.2025.1606224 (PMC12322897; doi:10.3389/fmed.2025.1606224)
Supplement: Supplementary file 1 [file Presentation_1.ppt]

## Slide 1
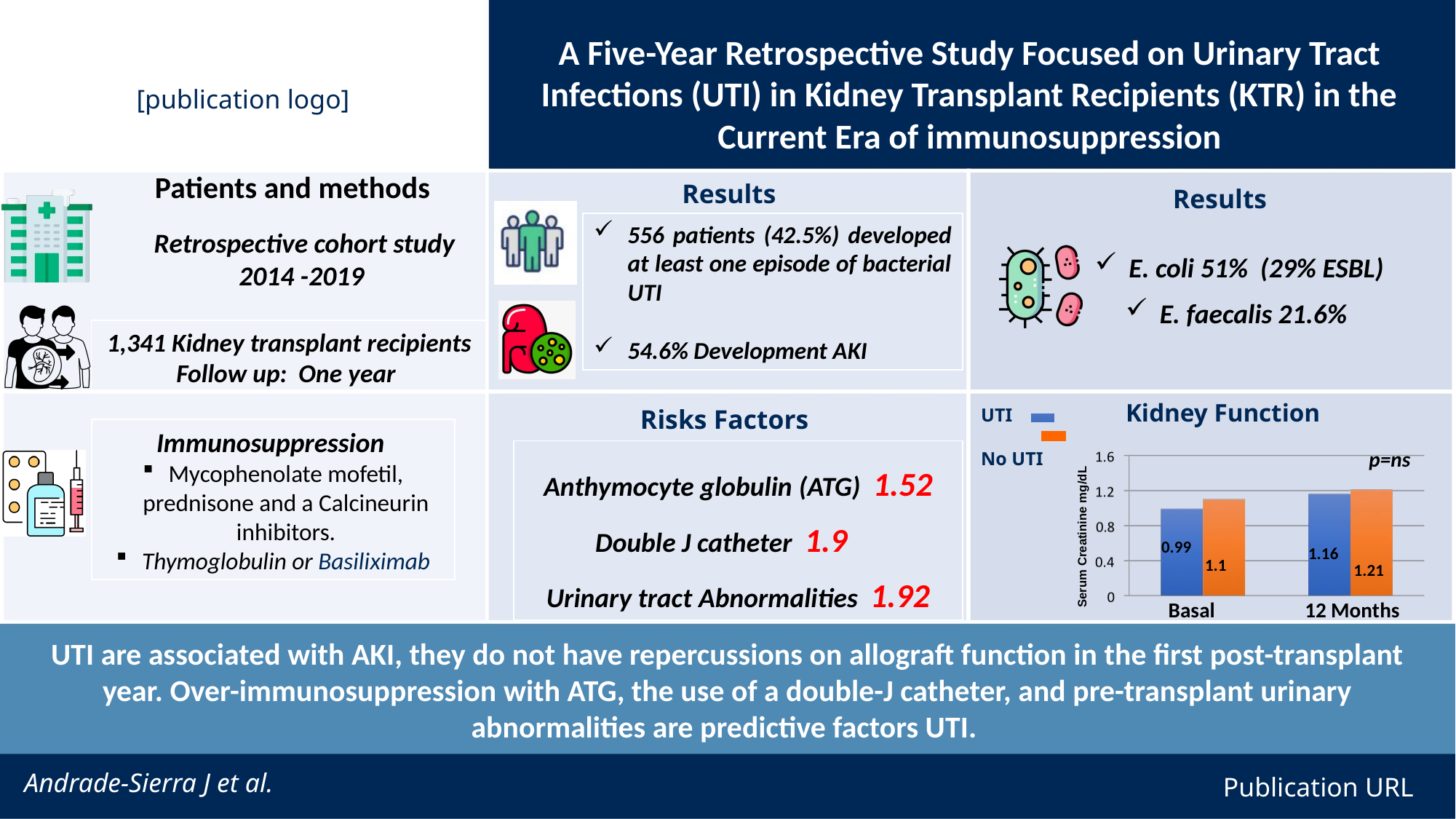

A Five-Year Retrospective Study Focused on Urinary Tract Infections (UTI) in Kidney Transplant Recipients (KTR) in the Current Era of immunosuppression
[publication logo]
Patients and methods
Results
Results
556 patients (42.5%) developed at least one episode of bacterial UTI
54.6% Development AKI
Retrospective cohort study
2014 -2019
E. coli 51% (29% ESBL)
E. faecalis 21.6%
1,341 Kidney transplant recipients
Follow up: One year
Kidney Function
Risks Factors
UTI
No UTI
Immunosuppression
Mycophenolate mofetil, prednisone and a Calcineurin inhibitors.
Thymoglobulin or Basiliximab
p=ns
Anthymocyte globulin (ATG) 1.52
Double J catheter 1.9
Urinary tract Abnormalities 1.92
Serum Creatinine mg/dL
0.99
1.16
1.1
1.21
Basal 12 Months
UTI are associated with AKI, they do not have repercussions on allograft function in the first post-transplant year. Over-immunosuppression with ATG, the use of a double-J catheter, and pre-transplant urinary abnormalities are predictive factors UTI.
Andrade-Sierra J et al.
Publication URL
